# Priority for radium therapy of benign conditions and cancer

**DOI:** 10.3747/co.2007.120

**Published:** 2007-06

**Authors:** R.F. Mould

**Keywords:** Radium, benign tumours, cancer, afterloading

## Abstract

In medicine, assigning priorities for original ideas and for first implementation of a new type of treatment or technology—radium afterloading, for example—is often difficult. This situation is certainly true for radium therapy, with conflicting claims coming from France, Germany, and the United States about who first implemented it. Moreover, if possible, a distinction must be made between the person who had the idea for a therapy and the person who actually implemented it. These people are not always one and the same. Difficulties in assigning priority also sometimes arise from the lack of a published claim in a medical journal, and extant photographic evidence is typically almost impossible to find some 100 years after the event. The present article tries to solve the problems of priority regarding those who were really responsible for the ideas and implementation of radium therapy, including the technique of afterloading.

## 1. INTRODUCTION

This article investigates priority claims for radium therapy with the aim of being able to cite literature sources in answering the following questions:

- Who first had the idea for radium therapy?
- Who first implemented radium therapy for benign skin diseases?
- Who first demonstrated a radium therapy cure for skin cancer?
- Who first proposed and implemented radium afterloading?

These questions are not as easy to answer as are those concerning the dates for the discovery of radioactivity by Becquerel and of radium by the Curies. What is even more difficult is to prioritize the use of radium therapy for specific body sites. Those priorities can usually be only a matter of guesswork from secondary sources such as textbooks on radiotherapy in the first decade of the twentieth century. The primary sources quoted are often in obscure journals that very few medical libraries possess—or they are not mentioned at all.

## 2. X-RAY THERAPY AS A PRECURSOR TO RADIUM THERAPY

The obvious precursor of radium therapy is X-ray therapy. Therapy using X-rays for skin conditions and diseases had already been demonstrated by the end of the nineteenth century, and unintentional skin burns had been observed.

Leopold Freund in Vienna is generally accepted as having been the first to use X-rays logically and scientifically within the limits of the age. His patient, a 5-year-old girl with a hairy nevus 36 cm in length, started treatment on November 24, 1896—X-rays having been discovered only 12 months earlier, on November 6, 1895. After successful epilation, an X-ray ulcer persisted for about 6 years, but it was considered to be cured in 1902 when only a scar remained. This patient was followed up when she was 75 years of age; apart from back pain, she was found to be in good health at that time 1–4. This epilatory effect of X-rays established them as a direct agent for producing biologic change.

Evidence for the harmful effects of X-rays had become known within a few months of their discovery. Thomas Edison 5 reported in March 1896 that his eyes were sore after he experimented with X-rays. Radiation burns to hands were reported 6 in the *British Medical Journal* in April 1896; and, in February 1897, in the *Bulletin of the Johns Hopkins Hospital,* a review 7 was given of 23 cases of X-ray injury reported in the literature before January 1897. Thus, by the time of the discoveries in 1898 of polonium 8 and radium 9 by Pierre and Marie Curie, the effects of X-rays on the skin were well known.

## 3. PRIORITY FOR RADIUM SELF-EXPOSURE EXPERIMENTS

### 3.1 Germany

The first two reports in the literature 10–12 of self-exposure experiments showing radium causing skin burns were made by two Germans, Friedrich Walkoff and Friedrich Giesel. In October 1900, Giesel described in a chemistry journal the strapping of 270 mg of radium salt to his inner forearm for a duration of 2 hours; he also wrote to inform Pierre Curie about the experiment. The Walkoff reference of 1900 consisted of just three lines in a three-page general review presented to a photographic club in Munich.

Giesel was the first to prepare radium sources in Germany for sale by the Buchler company of Braunschweig (Brunswick). In the first ten years of the twentieth century, this “German radium” competed commercially with “French radium,” such as that prepared and sold by Armet de Lisle in Paris. Interestingly, both companies were able to start radium production at an early date because both were involved in quinine production. Some of the manufacturing processes for quinine and radium were similar.

### 3.2 France

Henri Becquerel’s radium burn was accidental. He received it after he had placed a radium source inside his jacket pocket. However, he gave credit 13 for the first radium burn observations to Friedrich Walkoff and Friedrich Giesel.

Then, in June 1901, together with Pierre Curie, Becquerel reported 13 self-exposure measurements with radium using a source placed many times in “a pocket of his jacket.” Those experiments have been described in detail in *Current Oncology* 12.

### 3.3 United States

In the very early years following the discovery of radium, most studies took place in Europe. The United States and Canada lagged behind because radium sources were scarce, and no North American supplier had yet emerged. Most of the early sources obtained in the United States were brought back from Europe by visiting doctors and scientists. However, by about 1904, the United States was starting to catch up with Europe, and in Canada, Ernest Rutherford had commenced work at McGill University in Montreal.

In the United States, the reports of self-exposure experiments in 1904 by Robert Abbé, a New York surgeon, were the most detailed. Abbé performed radiobiologic experiments in 1904 14, and in 1907, he published 15 his answer to a friend’s question, “What difference is there between the light from the stars and radium rays?”

Abbé’s reply was as follows:

Radium rays move in absolute straight lines, without deviation by atmosphere, water, lenses or prisms, and nothing is opaque to them. They would even penetrate the stone column in my garden, which light does not. Using a small glass tube containing a bit of radium about the size of a grain of rice, 60 milligram pure radium bromide, German, and an exposure time of three days this is demonstrated. This same demonstration has been made by Röntgen rays, but it is far more impressive to witness the active energy incessantly given off by the innocent looking little tube of salt.

Abbé varied the exposure to radium in his experiments and correlated it with the degree of erythema and necrosis produced, publishing his results in the *New York Medical Record* 14,16,17.

George H. Stover of Denver, Colorado, was also among the first American physicians to obtain a supply of radium, purchasing some radium chloride in August 1903 from a Paris dealer. He was therefore one of the first Americans to conduct self-exposure experiments with radium, as reported in the *Colorado Medical Journal*:

My own experiments with this substance are as follows: The chloride of radium is enclosed in a sealed glass tube. On taking this tube between the fingers for from three to five minutes, I felt a distinct heat and tingling. Applying it to the skin of the forearm: After five to fifteen minutes the heat and tingling are felt. After half an hour this is noticed no longer, and the skin which has been directly under the tube is distinctly less sensitive to touch, pain and temperature, than the surrounding skin. After one hour to two hours there is left a slight erythema, which persists for some hours. 18

Stover was also a pioneer in the use of X-rays for diagnosis and therapy and was the first professor of Roentgenology at the Denver and Gross College of Medicine, and then of its successor institution, the University of Colorado School of Medicine. His early death was the result of excessive radiation exposure.

## 4. PRIORITY FOR RADIUM BRACHYTHERAPY

### 4.1 France

In 1996, a display cabinet in the Physics Department at the Ecole Supérieure de Physique et de Chimie Industrielles (espci) in the rue Vacquelin (formerly in 1898 part of the rue Lhomond) held a small collection of some of Pierre Curie’s apparatus and lecture notes. These items dated from when Curie worked at espci before, during, and after the discoveries of polonium and radium. A page dated November 29 and December 4 (without any year recorded) gave Pierre’s measurement of 0.394 g radium sulphate in a rubber-covered capsule for a person named Danlos.

**FIGURE 1:**
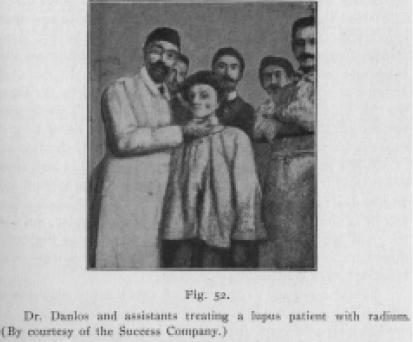
Photograph published in 1905 20 by Charles Baskerville, a chemist and mineralogist of New York. Baskerville described it as “Dr. Danlos and assistants treating a lupus patient with radium” and then continued in his text with “Danlos of St-Louis Hospital, Paris, apparently was the first to apply radium in the treatment of certain affectations of the skin similar to the treatment with Röntgen and the ultra-violet rays (Finsen).”

It has been known for many years that Henri Danlos of the Hôpital St-Louis, Paris, was the first physician to whom Pierre Curie loaned a source of radium. The first patient to receive treatment received it for lupus rather than for skin cancer, and a local surface application was reported in 1901 19 by Henri Danlos and Paul Bloch, but no photographs were included. However, a photograph of the treatment does exist (Figure 1) and was published in a 1905 book by Charles Baskerville 20. Baskerville described the dose as 19,000 X-units. This X-unit measurement was almost certainly the amount of radioactivity of the source as compared with an equal weight of uranium.

It should be noted, though, that Henri Danlos was not the first to have the idea of using radium to treat skin conditions. Louis Wickham and Paul Degrais, in the preface to their *Radiumtherapy* textbook of 1910 21, give the credit to Pierre Curie and to the Hôpital St-Louis dermatologist Ernest Besnier, who was 70 years old in 1901. Age would explain why Besnier did not carry out the treatment; Danlos was 57 years old and not retired. Ernest Besnier had earlier diagnosed Becquerel’s radium burn, which presumably gave him the idea that radium could be used to treat lupus 22.

### 4.2 United States

Danlos and Bloch published their results 19 in Paris in 1901, but in Boston, William Rollins had, in late 1900, given a radium source to his brother-in-law Francis Williams to be used for radium therapy. Francis Williams is therefore perhaps ahead of Henri Danlos in terms of priority for the clinical use of radium, even though the Boston results were not effective. The conditions treated by Williams with his weak source of radium were not recorded.

In the February 25, 1904, issue of the *Boston Medical and Surgical Journal* (the forerunner of the *New England Journal of Medicine*), Francis Williams wrote this:

Dr. William Rollins of Boston was, so far as I am aware, the first to appreciate that this new substance would probably be of service in the treatment of certain diseases. At least two or three years ago he put into my hands a metal box with an aluminium front, containing some chloride of radium, and at his suggestion I employed it for therapeutic purposes, but the radium to be had at that time was too weak to give definite results. 23

In 1908, Williams wrote again on the same topic as in 1904—a comparison between X-rays and radium—this time publishing in the *Journal of the American Medical Association* 24. He begins that paper with these words:

In 1900 Dr. William Rollins of Boston suggested the use of the radiations from radium salts as a therapeutic agent. The salts then available were not powerful enough to be efficient. To Dr. Rollins nevertheless, as far as I am aware, is due the credit of being the first to realize the probable value of these radiations as a therapeutic agent.

Williams also stated in his February 1904 paper 23 that he had recently treated 50 patients with pure radium bromide—usually 50 mg, but on some occasions varying between 10 mg and 100 mg. These cases consisted of “one [...] of acne, two of psoriasis, five of lupus vulgaris, two of lupus erythematosus, two of eczema, one of keloid, five of rodent ulcer, 28 of epidermoid carcinoma and four breast cases.”

### 4.3 Germany

In Germany, it appears that Hermann Strebel of Munich might have had priority ahead of Henri Danlos for being the first to use radium clinically, but as in the case of Francis Williams, the results were not effective. He also was probably the first to whom it occurred to actually use radium for therapy, preceding Ernest Besnier. Strebel’s report 25 in 1903 in the *Deutsche Medizinische Zeitung* stated that he “placed a quantity of radium enclosed in a capsule made from cardboard and paraffined paper on an area of lupus and left it in place for hours. A moderate reaction was obtained with subsequent fading of the lupus node. With further irradiation an ulcer formed which healed only poorly, but the lupus was not healed.” Strebel reasoned that the unsatisfactory results were attributable to “low radiation capacity of the radium available.” He then noted that Danlos and Bloch had announced their successful treatment of lupus vulgaris *after* his own unsuccessful attempts.

### 4.4 Russia

Non-malignant skin conditions such as lupus and angioma were chosen for treatment before skin cancers. Then, in 1903 in St. Petersburg, reporting 26 in the *Dermatologische Zeitschrift,* S.W. Goldberg and Efim Semenovich London claimed the first histologically proven cure of a basal cell carcinoma of the face. However, unlike the first documented 27,28 histologic cures from X-rays (also for basal cell carcinomas of the face, in 1899 in Stockholm), no photographs of the radium patient before and after treatment are known.

## 5. PRIORITY FOR RADIUM AFTERLOADING

In 1903, Hermann Strebel of Munich was the first person to propose 25 the use of afterloading, although Robert Abbé of New York who published in 1906 15 is often given priority for this important radium brachytherapy technology proposal.

In his report, Strebel comments that the radium sources available to him in 1903 had a strength of 1.5 million uranium units and were capable of producing radiation dermatitis lasting 8–14 days after an exposure of only 10 minutes. It was to avoid this problem that he instituted his interstitial afterloading technique:

I am now in a position to increase the effectiveness of radium for deeper seated pathological conditions quite significantly, without causing undesirable effects on the skin itself. Instead of surface applications, intratumoural application is carried out by inserting the radium, which is enclosed in the drilled tip of a small aluminium rod, directly into the centre of the tumour, with the help of a previously inserted trepan. This increases the size of the irradiation beam in that the radium irradiates evenly in all directions. 25

Strebel then continued to advocate the trepan (afterloading guide) needle being inserted in various directions, always using the first insertion opening, and he stated that this method is suitable for cancers of the uterus, stomach, and liver, and “can be used successfully as a substitute for X-rays when it is a question of the treatment taking place within small spaces such as the nose, larynx, and bladder.”

**FIGURE 2:**
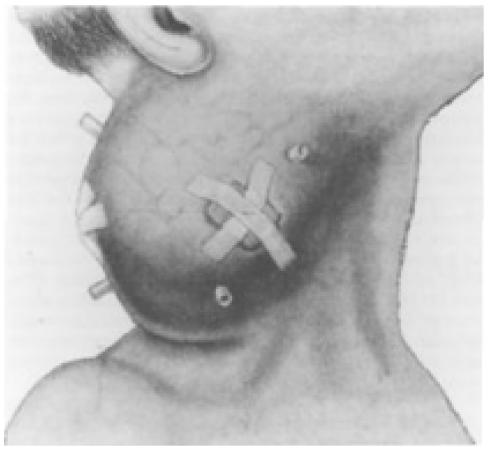
An interstitial technique using crossfire for the treatment of an inoperable malignant tumour in the neck 29.

The idea of insertion of the radium within the trepan needle in various directions is essentially the idea of crossfire irradiation, although Strebel did not use that term. It was left to Louis Wickham and Paul Degrais in 1910 21 to clearly define crossfire. In 1913, they further described an interstitial technique for the treatment of an inoperable malignant tumour of the neck in which they used goose quills as afterloading guides (Figure 2) 29. They reported that the quills “allowed introduction of radium tubes deep into tissues at four opposite points of the tumour.”
